# *HFE*-Related Hemochromatosis: The Haptoglobin 2-2 Type Has a Significant but Limited Influence on Phenotypic Expression of the Predominant p.C282Y Homozygous Genotype

**DOI:** 10.1155/2009/251701

**Published:** 2009-11-05

**Authors:** Gérald Le Gac, Chandran Ka, Isabelle Gourlaouen, Laurence Bryckaert, Anne-Yvonne Mercier, Brigitte Chanu, Virginie Scotet, Claude Férec

**Affiliations:** ^1^Etablissement Français du Sang Bretagne, Brest 29200, France; ^2^Inserm, U613, Brest 29200, France; ^3^Laboratoire de Génétique Moléculaire et d'Histocompatibilité, Hôpital Morvan, Centre Hospitalier Universitaire de Brest, Brest 29200, France; ^4^Faculté de Médecine de Brest et des Sciences de la Santé, Université de Bretagne Occidentale, Brest 29200, France

## Abstract

Phenotypic expression of the common p.C282Y/p.C282Y *HFE*-related hemochromatosis genotype is heterogeneous and depends on a complex interplay of genetic and non-genetic factors. Haptoglobin has a crucial role in free hemoglobin iron recovery, and exists as three major types: Hp1-1, Hp2-1 and Hp2-2. Hp2-2 favors endocytosis of hemoglobin iron in monocytes/macrophages, resulting in partial iron retention and increased intracellular ferritin levels. This situation is generally not expected to severely affect iron homeostasis, but was found to correlate with elevated serum iron indices in healthy men. Whether the Hp2-2 genotype acts as a modifier in *HFE*-related hemochromatosis is unclear. In this study we investigated influence of Hp2-2 and of potential confounders on the iron indices of 351 p.C282Y homozygous patients. We conclude that there is a cause-and-effect relationship between the Hp2-2 genotype and increased iron indices in p.C282Y homozygous patients. The Hp2-2 effect is, however, limited and only apparent in males.

## 1. Introduction

Hemochromatosis (HC; synonymous with hereditary hemochromatosis) refers to several inherited disorders of iron metabolism characterized by progressive iron accumulation in major organs [1–4]. If not recognized and treated, this progressive iron loading can lead to tissue damage and severe clinical complications, including cirrhosis, hepatocellular carcinoma, and cardiomyopathy [5].

The historical and predominant form of hemochromatosis is an adult-onset autosomal recessive condition usually associated with the *HFE* p.C282Y/p.C282Y genotype. In Northern European populations, this genotype is carried by approximately 1 person in 200 [4]. However, its phenotypic expression is heterogeneous and clearly depends on a balance between accentuating and reducing factors. The influence of gender, age, and lifestyle components, such as alcohol abuse, has been well documented [2, 6, 7]. Involvement of modifier genes or epigenetic mechanisms is less obvious, but is supported by studies in *Hfe* knockout mice, as well as by the greater concordance between biological and clinical iron overload phenotypes within families than between families [4, 8, 9].

Most iron in the body is directed to erythropoiesis and is present as heme in hemoglobin (Hb). Hemoglobin iron is normally recycled after destruction of senescent erythrocytes by macrophages. Under physiological conditions, however, up to 10% of Hb molecules escape into the plasma [10]. These free Hb molecules are bound by haptoglobin (Hp), which delivers them to the hepatic parenchymal cells, Kupffer cells and other macrophages. In this way, haptoglobin prevents iron losses through the glomeruli and protects the kidney against oxidative injury [11].

Haptoglobin is synthesized as a single polypeptide chain, which is cleaved in the endoplasmic reticulum to an amino-terminal *α*-chain and a carboxy-terminal *β*-chain [12]. The basic structure of Hp is a homodimer designated Hp1-1 in which the two Hp molecules are joined by a single disulfide bridge through their respective *α*-chains. Humans differ from other species by having two common *Hp* alleles (denoted *Hp1* and *Hp2*), which, due to partial gene duplication, code for either a short or a long *α*-chain, and the *β*-chain. Since the elongated *α*-chain contains two cysteine residues that form disulfide bridges with two other *α*-chains, individuals homozygous for the *Hp2* allele show a multimeric Hp type designated Hp2-2. Hp2-1 refers to the type (both Hp dimers and multimers) seen in individuals heterozygous for the two *Hp* alleles [13].

Hp2-2 has been reported to favor endocytosis of Hb-Hp complexes by monocytes/macrophages and to produce partial iron retention [13, 14]. This results in higher intracellular ferritin concentrations, whereas the impact on serum iron parameters was found to be moderate and to reach significance only in healthy males [14]. In agreement with this gender-related effect, Hp2-2 was associated with elevated serum iron and ferritin levels in p.C282Y homozygous male patients but not in p.C282Y homozygous female patients [15]. However, this finding has been challenged by two other studies [16, 17].

Tolosano et al. have recently reported that *Hfe* and *Hp* compound knockout mice accumulated significantly less hepatic iron than *Hfe*-null mice, thus demonstrating that Hp-mediated Hb-iron recovery may contribute to iron loading in HC [18]. *Hp2* does not designate a null allele. Rather, it encodes a protein that could favor free hemoglobin iron delocalization into macrophages. To gain insight into the role of *Hp* as a modifier gene in *HFE*-related hemochromatosis, we investigated the influence of the Hp2-2 genotype in a large number of p.C282Y homozygous patients. We hypothesized that it might be limited and masked by the predominant effect of gender and other important non-genetic modifiers (age and alcohol abuse).

## 2. Materials

### 2.1. Study Patients

Three-hundred and fifty-one patients were enrolled in the study. At the first visit of these patients to a healthcare center in Brittany (western part of France), a clinical questionnaire was completed by the specialized physician. Information contained in this questionnaire was previously described in detail [6]. It provided information regarding sociodemographic characteristics of patients, their age at diagnosis, the circumstances of HC diagnosis, the biochemical parameters, and the clinical signs associated at the time of onset.

Basic reasons for diagnosis were presence of the *HFE* p.C282Y/p.C282Y genotype and the observation of abnormal iron indices (elevated transferrin saturation with or without elevated serum ferritin concentration). It means that not all patients presented with clinical signs.

The selected patients did not carry additional mutations on *HAMP* and *HJV*, two previously reported modifiers of the p.C282Y homozygous phenotype [19, 20].

This study complied with French bioethics regulations. Informed consent of patients was obtained before blood samples were taken.

### 2.2. Assessment of the Amount of Iron Removed by Phlebotomy and of Daily Alcohol Consumption

All patients were followed up in the same healthcare center, under regular practices (the targeted value for serum ferritin was 100 *μ*g/L).

The amount of iron removed by phlebotomy was assessed from the volume of blood extracted during depletion therapy, assuming that 500 mL of blood contains 250 mg of elemental iron. A total of 26 patients (17 males and 9 females) had not completed iron depletion therapy when the study began. This subgroup of patients was excluded from all statistical analyses of the iron removed by phlebotomy variable.

Daily alcohol consumption was assessed from a detailed item included in the clinical questionnaire, which measured the amount of beer, wine, and liquor consumed, assuming respective alcohol (ethanol) contents of 4%, 12%, and 20%. Alcohol abuse was defined as consumption of ≥60 g per day [21].

### 2.3. Haptoglobin Genotyping

Haptoglobin polymorphism was determined using two complementary allele-specific PCRs, as fully described by Koch and collaborators [22]. Briefly, a first PCR exploits the size difference due to the two extra exons of the *Hp2* allele. It leads to the amplification of a 1757-bp product from the *Hp1* allele and of a 3481-bp product from the *Hp2* allele. Because of the primer binding sites and their 5′ to 3′ orientations, a second PCR permits the amplification of a 349-bp product from the *Hp2* allele but no amplification from the *Hp1* allele. This makes identification of the Hp2-1 heterozygous genotype more reliable as, in the first round of PCR, amplification of the *Hp1*-specific 1757-bp product is more efficient and occasionally leads to a very weak detection of the Hp2-specific 3481-bp product.

### 2.4. Statistical Analyses

Statistical analyses were performed using Epi-Info (version 6.04; Centers for Disease Control and Prevention, Atlanta, Ga, USA) or SAS (version 9.1; SAS Institute, Cary, NC) software.

Analysis of variances (ANOVA) and Student's *t*-test, considering either the pooled (equal variance) or the Satterthwaite (unequal variance) method, were used to test for between-group differences in means. Logarithmic transformation were used for all statistical analyses to ensure that serum ferrtin and iron removed by phlebotomy were approximately normally distributed.

The following potential confounders were considered in multiple linear regression models with either serum ferritin concentration or amount of iron removed by phlebotomy as dependent variable: *Hp* genotypes, gender, age at diagnosis (three dummy variables defined by the quartiles with quartile 1 as reference), and alcohol consumption (abusers versus non abusers). *Hp* genotype variable was assessed by grouping the Hp1-1 and Hp2-1 genotypes, and by only considering the Hp2-2 genotype as potential predictor. This allowed us to increase the size of the category used as reference. It should be noted that the *Hp* genotype variable was initially coded as two dummy variables (Hp2-1 versus Hp1-1 and Hp2-2 versus Hp1-1), and that by contrast with Hp2-2, Hp2-1 did not show any association with higher serum ferritin or iron removed by phlebotomy.

As serum ferritin concentration and iron removed by phlebotomy were log_10_ transformed, the regression coefficient *β* corresponded to a 10^*β*^ times change in mean Y (i.e., serum ferritin or iron removed) per unit increment in the explanatory variable. In a multiple linear regression model, the coefficients *β* are ajusted for the other covariables.”

## 3. Results

### 3.1. Hp Genotype Distribution

Hp genotypes among the 351 p.C282Y homozygous were distributed as follows: 46 for Hp1-1 (13.1%), 179 for Hp2-1 (51.0%), and 126 for Hp2-2 (35.9%). These genotype frequencies were in accordance with previous reports in Northern European populations [15, 17, 23].

### 3.2. Iron Overload Status Regarding Hp Genotypes

#### 3.2.1. Univariate Analyses

Analysis of variance failed to reveal any significant difference between the various *Hp *groups of p.C282Y homozygous patients, in terms of age at diagnosis, transferrin saturation, log-transformed serum ferritin, and log-transformed iron removed by phlebotomy (Table 1(a)).

These results contrasted with the positive effects of male sex and alcohol abuse on all the studied iron parameters (Tables 1(b) and 1(c); Student's *t*-tests).

#### 3.2.2. Multivariate Analyses

Multiple linear regression models were fitted to test influence of *Hp* genotypes (Hp2-2 versus Hp1-1 and Hp2-1) on either log-transformed serum ferritin or log-transformed iron removed by phlebotomy, after adjustment for gender, age at diagnosis, and alcohol consumption (Table 2). For both the dependent variables assessed, higher values were not only seen in male patients (*P* < .0001 and *P* < .0001, resp.), patients of greater age at diagnosis (*P* < .0001 and *P* < .0001) and patients who drank at least 60 g of alcohol per day (*P* = .0073 and *P* = .0096) but also in patients with the Hp2-2 genotype (*P* = .0033 and *P* = .0024).

With log_10_transformation of the dependant variable (i.e. serum ferritin), the regression coefficient of the “Hp group” variable was 0.117. It means that average serum ferritin was 10^0.117^ or 1.31 times higher in patients with Hp2-2 genotype than in patients with Hp1-1 or Hp1-2 genotype, this after adjustment on other covariables. The model also indicated that mean serum ferritin concentration appeared 1.57 times higher in alcohol abusers that in non alcohol abusers.

We did not observed an interaction between gender and Hp genotype (*P* = .5959). However, because gender was confirmed as the major modifier in phenotypic expression of the p.C282Y homozygous genotype, and because the effect of the Hp2-2 genotype on serum iron parameters was previously restricted to the male sex [14, 15], analyses were performed in men and women, separately*.*

### 3.3. Iron Overload Status Regarding Gender and Hp Genotypes

As only two of 27 heavy alcohol drinkers were females, patients who drank less than 60 g of alcohol per day were first considered. In this category, analysis of variance revealed significant differences in means of log-transformed serum ferritin and log-transformed iron removed by phlebotomy between the various *Hp* groups of males, but not of females (data not shown). As expected, subsequent pairwise *t*-tests ascribed the observed differences to higher serum ferritin and higher iron removed by phlebotomy in male carriers of the Hp2-2 genotype (data not shown).

Linear regression analysis indicated that these observations remained unchanged after adjustment for age at diagnosis and daily alcohol consumption (Tables 3(a) and 3(b)).

## 4. Discussion

Only a small fraction of hemoglobin iron passes through the haptoglobin recovery pathway. For this reason, it is generally believed that Hp polymorphism does not severely affect iron homeostasis, and does not consistently explain the large phenotypic variability seen in carriers of the common *HFE* p.C282Y/p.C282Y genotype. However, Vlierberghe et al. have reported a striking association between Hp2-2 and more pronounced iron overload phenotypes in p.C282Y/p.C282Y males [15]. In view of this finding, and also bearing in mind contradictory findings [16, 17], our study underlines that the contribution of the Hp2-2 type is significant but limited. It is only apparent when considering gender and is further found to be moderate in comparison to age and alcohol abuse (as evidenced by regression coefficients in Table 2).

Lack of replication between earlier investigations of the influence of the Hp2-2 type on phenotypic expression of the p.C282Y homozygous genotype may have several explanations. It notably might reflect differences in selection and size of the sample studied. Here, we enrolled a large number of expressive p.C282Y homozygous individuals; that is, individuals who displayed, at least, biological changes of the disease (increased transferrin saturation levels, with or without increased serum ferritin concentrations). This permitted powerful statistical analyses and careful evaluation of the relationship between the Hp2-2 genotype and the severity of iron overload. In this way, our results were similar to those initially reported by Vlierberghe et al. from a cohort of 167 p.C282Y homozygous patients [15]. In the overall population of patients, univariate analyses revealed that all the studied iron parameters were slightly increased in carriers of the Hp2-2 genotype. Stratification of the population by gender was, however, needed to obtain significant differences. These differences were restricted to the male subgroup, and to markers of body iron excess.

As *HFE*-related hemochromatosis develops slowly, gender and age are recognized as important modifying factors [3]. Menstruation and pregnancy, which deplete iron stores, act as temporary protective factors in women of reproductive age, whereas biological changes and subsequent clinical manifestations of the disease are commonly less marked in younger people than in older people. In addition, alcohol intake is known to worsen the hepatic damage produced by iron overload [21, 24]. We have previously shown that alcohol abuse (alcohol consumption >60 g per day) results in higher iron parameters, and more frequent clinical manifestations [6]. To permit a more incisive examination of whether there is a true cause-and-effect relationship between the Hp2-2 genotype and increased iron parameters, we fitted multiple linear regression models with gender, but also age at diagnosis and alcohol abuse as potential confounders. These models led to the presentation of the Hp2-2 genotype as an independent factor increasing serum ferritin concentrations and iron removed by phlebotomy (Table 2). However, the models also showed that Hp2-2 was the lowest predictor. Adjusting for the strong effect of sex and age, carriers of the Hp2-2 genotype were thus less likely than alcohol abusers to have elevated serum ferritin and iron removed by phlebotomy. This latter point was verified in specific tests where medians for serum ferritin and iron removed by phlebotomy were significantly higher in alcohol abusers (who carried the Hp1-1 or Hp1-2 genotype) than in patients with the Hp2-2 genotype (who declared no excessive alcohol intake). The observed values were 1256.0 *μ*g/L [Q1 = 803.0, Q3 = 2311.0] versus 599.0 *μ*g/L [Q1 = 319.5; Q3 = 975.0] (*P* = .0010) and 6.6 g [Q1 = 4.8; Q3 = 9.7] versus 3.6 g [Q1 = 1.8; Q3 = 6.8] (*P* = .0055), repectively. 

We conclude that, by accentuating the iron burden, the frequent Hp2-2 genotype could explain some of the phenotypic heterogeneity seen in p.C282Y homozygous hemochromatosis patients. However, we support the idea that this influence is weak, and that other genetic and environmental modifying factors should be considered, to efficiently distinguish those homozygous patients at higher risk of developing morbid complications.

## Figures and Tables

**Table:** (a) Sociodemographic and biochemical characteristics of the 351 p.C282Y homozygous patients according to *Hp* genotype.

Characteristic	*Hp* genotype			
	Hp1-1	Hp2-1	Hp2-2	*P* value
Sociodemographic				
Number of patients	46	179	126	
Gender (Males, Females)	32 M, 14 F	99 M, 80 F	64 M, 62 F	
Age at diagnosis (years)	46.5 ± 10.8	47.4 ± 13.8	46.4 ± 13.8	0.7887
Biochemical				
Transferrin saturation (%)	76.8 ± 15.1	78.7 ± 14.3	80.0 ± 12.9	0.4009
Serum ferritin (*μ*g/L)	604 [310–1136]	570 [280–1024]	631 [329–1196]	0.3580
Iron removed (g)^1^	3.6 [1.8–6.1]	3.3 [1.8–5.6]	3.9 [2.0.–6.8]	0.3030

**Table:** (b) Sociodemographic and biochemical characteristics of the studied 351 p.C282Y homozygous patients according to gender.

Characteristic	Gender		
	Males	Females	*P* value
Sociodemographic			
Number of patients	195	156	
Age at diagnosis (years)	46.5 ± 12.5	47.5 ± 14.5	0.4854
Biochemical			
Transferrin saturation (%)	82.0 ± 13.1	75.0 ± 14.0	<0.0001
Serum ferritin (*μ*g/L)	892 [553–1732]	320 [179–562]	<0.0001
Iron removed (g)^1^	4.9 [3.4–8.2]	3.2 [2.0–5.8]	<0.0001

**Table:** (c) Sociodemographic and biochemical characteristics of the studied 351 p.C282Y homozygous patients according to alcohol consumption.

Characteristic	Alcohol consumption		
	≥60 g/day	<60 g/day	*P* value
Sociodemographic			
Number of patients	27	324	
Gender (Males, Females)	25 M, 2 F	170 M, 154 F	
Age at diagnosis (years)	48.9 ± 12.5	46.7 ± 13.5	0.4185
Biochemical			
Transferrin saturation (%)	88.3 ± 8.0	78.1 ± 14.0	0.0002
Serum ferritin (*μ*g/L)	1256 [658–2315]	562 [283–967]	<0.0001
Iron removed (g)^1^	6.8 [4.8–9.7]	3.4 [1.8–5.9]	<0.0001

^1^ *N* = 325 (178 males and 147 females).

Age at diagnosis and transferrin saturation are expressed as mean ± standard deviation.

Serum ferritin concentrations and iron removed by phlebotomy are presented as median and interquartile range.

**Table 2:** Regression coefficients (*β*) and 95% confidence intervals (CIs) for variables influencing serum ferritin (*μ*g/L; log_10_ transformed) and iron removed by phlebotomy (g; log_10_ transformed) in p.C282Y homozygous patients.

Variable	Serum ferritin^1^		Iron removed^2^	
	10^*β*^ (95% CI)	*P* value	10^*β*^ (95% CI)	*P* value
Gender^3^	0.33 (0.27, 0.39)	<0.0001	0.38 (0.33, 0.45)	<0.0001
Age at diagnosis^4^				
Quartile 2	1.33 (1.05, 1.69)	0.0204	1.27 (1.02, 1.58)	0.0304
Quartile 3	1.77 (1.39, 2.26)	<0.0001	1.48 (1.19, 1.84)	0.0005
Quartile 4	1.88 (1.47, 2.39)	<0.0001	1.63 (1.30, 2.03)	<0.0001
Alcohol consumption^5^	1.57 (1.13, 2.18)	0.0073	1.53 (1.11, 2.12)	0.0096
Hp group^6^	1.31 (1.09, 1.57)	0.0033	1.29 (1.09, 1.52)	0.0024

^1^ *N* = 351 (195 males and 156 females). Adjusted *r* ^2^ = 38.3%.

^2^ *N* = 325 (178 males and 147 females). Adjusted *r* ^2^ = 36.1%.

^3^Male sex was used as the reference category.

^4^Quartile 1 was used as the reference category.

^5^Daily alcohol intake < 60 g was used as the reference category.

^6^Hp1-1 and Hp2-1 genotypes were grouped and used as the reference category.

**Table:** (a) Regression coefficients (*β*) and 95% confidence intervals (CIs) for variables influencing serum ferritin (*μ*g/L; log_10_-transformed) and iron removed by phlebotomy (g; log_10_-transformed) in p.C282Y homozygous males patients.

Variable	Serum ferritin^1^		Iron removed^2^	
	10^*β*^ (95% CI)	*P* value	10^*β*^ (95% CI)	*P* value
Age at diagnosis^3^				
Quartile 2	1.30 (0.99, 1.71)	0.0603	1.29 (0.99, 1.66)	0.0567
Quartile 3	1.87 (1.39, 2.51)	<0.0001	1.39 (1.05, 1.68)	0.0204
Quartile 4	1.59 (1.19, 2.13)	0.0019	1.50 (1.15, 1.96)	0.0032
Alcohol consumption^4^	1.59 (1.18, 2.16)	0.0027	1.50 (1.11, 2.04)	0.0096
Hp group^5^	1.40 (1.13, 1.74)	0.0026	1.38 (1.12, 1.69)	0.0022

**Table:** (b) Regression coefficients (*β*) and 95% confidence intervals (CIs) for variables influencing serum ferritin (*μ*g/L; log_10_-transformed) and iron removed by phlebotomy (g; log_10_-transformed) in p.C282Y homozygous females patients.

Variable	Serum ferritin^1^		Iron removed^2^	
	10^*β*^ (95% CI)	*P* value	10^*β*^ (95% CI)	*P* value
Age at diagnosis^3^				
Quartile 2	1.26 (0.83, 1.89)	0.2723	1.31 (0.90, 1.91)	0.1574
Quartile 3	1.85 (1.23, 2.78)	0.0033	1.58 (1.09, 2.29)	0.0154
Quartile 4	2.38 (1.58, 3.59)	<0.0001	1.87 (1.29, 2.71)	0.0012
Alcohol consumption^4^	1.37 (0.37, 5.01)	0.6351	1.65 (0.53, 5.15)	0.3892
Hp group^5^	1.20 (0.89, 1.61)	0.2383	1.17 (0.90, 1.53)	0.2457

^1^ *N* = 195. Adjusted r^2^ = 16.2%.

^2^ *N* = 178. Adjusted r^2^ = 13.5%.

^3 ^Quartile 1 was used as the reference category.

^4 ^Daily alcohol intake < 60 g was used as the reference category.

^5 ^Hp1-1 and Hp2-1 genotypes were grouped and used as the reference category.
